# Phytochemical Composition and Biological Activities of Wild *Scolymus maculatus* L.

**DOI:** 10.3390/medicines6020053

**Published:** 2019-04-30

**Authors:** Saleh Abu-Lafi, Mahmoud Rayan, Mahmud Masalha, Basheer Abu-Farich, Hashem Al-Jaas, Malek Abu-Lafi, Anwar Rayan

**Affiliations:** 1 Faculty of Pharmacy, Al-Quds University, Abu-Dies 144, Palestine; sabulafi@staff.alquds.edu; 2 QRC—Qasemi Research Center, Al-Qasemi Academic College, Baka El-Ghrabiah 30100, Israel; mahmoud_ryan@hotmail.com (M.R.); mahmudmasalha@gmail.com (M.M.); af_basheer@qsm.ac.il (B.A.-F.); 3 Central Public Health Laboratory, Ministry of Health, Ramallah 4284, Palestine; hashem_222@yahoo.com; 4 Faculty of Medicine, Al-Quds University, Abu-Dies 144, Palestine; Malek.al.1991@gmail.com; 5 Drug Discovery Informatics Lab, the Institute of Applied Research - Galilee Society, Shefa-Amr 20200, Israel

**Keywords:** *Scolymus maculatus*, phytochemicals, GC-MS analysis, herbal medicine, free radical scavenging, antioxidant, antimicrobial, DPPH assay

## Abstract

**Background:** The wild population of spotted golden thistle, *Scolymus maculatus*, which belongs to the *Compositae* family, is believed to be one of the multi-curative wild plants mentioned in *Flora Palaestina*. This study aims to disclose the phytochemical composition, antioxidant potential, and antimicrobial activity of wild *S. maculatus* collected from the farms of Kabul, a village in northwest Galilee, for the first time. **Methods:** The phytochemical components of crude *S. maculatus* extracts from methanol, ethyl acetate, and *n*-hexane solvents were separated and identified using gas chromatography-mass spectrometry (GC-MS) in the electron impact (EI) mode. The free radical scavenging of the plant extracts was measured by DPPH assay. The microdilution test was used to determine the minimum inhibitory concentrations (MICs) of different *S. maculatus* extracts and to evaluate their antimicrobial activities. **Results:** Thirty-two phytochemicals were found in *S. maculatus* extracts including stigmasterol, γ-sitosterol, lupeol, lupeol acetate, and β-amyrin. Phytochemicals, such as 2-linoleoylglycerol, γ-sitosterol, β-amyrin, lupeol, (3α)-12-oleanen-3-yl acetate, and lupenyl acetate, were found to dominate the methanol extract. Most of these compounds were also observed in ethyl acetate and *n*-hexane extracts, but at different levels, in addition to some other minor compounds. The various extracts were investigated for their antioxidant and antimicrobial activity. The ethanolic and the methanolic extracts were shown to exhibit the highest free radical scavenging by DPPH assay with a half-maximally effective concentration (EC_50_) of 0.37 and 0.65 mg/mL respectively, while the other three extracts (aqueous, ethyl acetate and *n*-hexane) were less active and their EC_50_ (effective concentration at which DPPH radical was scavenged by 50%) were above 1.0 mg/mL. Moreover, MICs were determined to be effective against *Staphylococcus aureus*, *Salmonella typhimurium*, and *Candida albicans* microorganisms. Ethyl acetate and the ethanolic extracts are active against the three types of microorganisms at a minimum inhibitory concentration (MIC) of 0.5 mg/mL, while aqueous and the *n*-hexane extracts are inactive against *Salmonella typhimurium*. **Conclusions:** The results show that *S. maculatus* extracts are a rich source of compounds that can play an important role in human health, and in a broader context, in the treatment of various diseases, such antimicrobial and antioxidant-related ailments.

## 1. Introduction

Plant-based nutrients and natural products have long been considered to be linked with human health and even evidenced to reduce risks of chronic human illnesses, such as inflammation [1,2,3], diabetes [4], cancer [5,6], and microbial-related diseases [7,8,9,10,11]. To date, herbal medicine is widely practiced in Palestine [12]. Well-reputed, it plays an integral part in the cultural heritage and public healthcare practices of the region. A paucity of herbal products used in folk medicine has been scientifically investigated and recorded [13,14]. The efficacy, safety, toxicity, dosage, and the usage instructions for medicinal plants are generally transmitted verbally from one generation to another [14]. One of the putative multi-curative wild herbal plants of *Flora Palaestina* is the spotted golden thistle, *S. maculatus* (*Compositae* family). This wild plant is very common in the Mediterranean region [15]. Its shape is very similar to the famous milk thistle plant, *Silybum marianum* (L.), which is used to protect the liver. S. *marianum*’s main active ingredients are silybin, silydianin, and silychristine, collectively known as *silymarin* [15]. *S. maculatus*, however, is also believed to contain other useful phytochemicals that may prevent and treat diseases, such as liver disease, cancer, and diabetes. Moreover, a stem decoction is traditionally prepared to treat intestinal and kidney inflammation [15]. In light of this, wild *S. maculatus* phytochemicals from methanol, ethyl acetate, and *n*-hexane solvents were investigated using gas chromatography-mass spectrometry (GC-MS) in the electron impact (EI) mode to identify authentic principal compounds. Thirty-two phytochemicals were detected for the first time in S. *maculatus*. Moreover, in vitro antioxidant activities were tested using DPPH assay. MICs were determined for effectiveness against *Staphylococcus aureus (S. aureus)**,** Salmonella typhimurium*, and *Candida albicans* microorganisms.

## 2. Materials and Methods

### 2.1. Plant Collection and Extract Preparation

Whole *S. maculatus* plants, comprising stems and flowers, were collected from the farms of the village of Kabul in the northwest of Galilee during October 2014. The plant was identified and authenticated by Dr. Khaled Sawalha, a botanist from the Biology Department of Al-Quds University. The specimens were washed with distilled water and dried in the shade for three weeks. Two hundred milliliters each of water, methanol, ethanol, ethyl acetate, and *n*-hexane were added separately to the dried ground plant material (20 g) in a beaker, and all the samples were sonicated for 60 min at 45 °C, then left in a dark glass bottle for 24 h for complete extraction. The extracts were filtered by passing the solvents through a 0.2 µm filter. Ten milliliters were taken from the extracts for GC-MS tests. The solvent remaining from each extract was evaporated with a rotary vacuum evaporator under reduced pressure. The yields of the extracts (*w*/*w*%) were 3.3%, 3.7%, 2.4%, 7.9%, and 2.3% for aqueous, methanolic, ethanolic, ethyl acetate, and *n*-hexane, respectively. The crude extracts were dissolved in DMSO to prepare stock solutions with a 16.0 mg/mL concentration, then subjected to free radical scavenging tests using DPPH assay. Microdilution tests were conducted to determine MICs of the extracts against *Staphylococcus aureus*, *Salmonella typhimurium*, and *Candida albicans* microorganisms. The chemical compositions of the three extracts were verified by GC-MS.

### 2.2. Instrumentation

The extracts were analyzed using a Perkin Elmer, Clarus gas chromatograph connected to a Clarus 600 C mass spectrometer (GC-MSPerkinElmer, Inc, Shelton, CT, USA). The GC-MS was operated in the electron impact ionization mode (EI) at 70 eV. A Perkin Elmer autosampler was used with 2 mL vials. The capillary GC column (DB-5 MS, Agilent Technologies, Inc, Santa Clara, CA, USA) was equipped with fused silica that consisted of 5% diphenyl polysiloxane and 95% dimethyl polysiloxane, 28 m × 0.25 mm, with a coating film thickness of 0.25 µm (Restek Corporation, Bellefonte, PA, USA). 

### 2.3. GC-MS Chromatographic Condition

The flow rate of the carrier gas was 1 mL He/min. The injector temperature was set at 235 °C, the source temperature at 250 °C, and the interface temperature at 260 °C. A split ratio of 1:20 was adopted during the entire analysis. The column gradient temperature was held at 50 °C for two minutes, then raised from 50 °C to 180 °C at a ramp rate of 5 °C/min and from 180 °C to 280 °C at a ramp rate of 15 °C/min and held there for an extra five minutes. A solvent cut time of 4.5 min was used to eliminate the solvent’s gigantic peak. The mass range was from 50 up to 480 Da, with a scan interval of 0.2 seconds. The identification of compounds was mainly based on matching their MS spectra with those of NIST mass spectral library.

### 2.4. Free Radical Scavenging Capabilities of Extracts

The free radical scavenging of different concentrations of the *S. maculatus* plant extracts was measured by DPPH assay [16]. One-milliliter portions of the plant extract were added to one-milliliter ethanolic DPPH solution (with a concentration of 100 ppm). The assay was performed using two-fold serial dilution by DMSO, starting from the stock solution of the plant extract. The mixture was shaken strongly and allowed to stand for 30 min at room temperature in a dark place. The absorbance of the solution was measured at 517 nm and converted into a percentage of free radical scavenging (FRS%) using the following equation:

FRS% = 100 × {1 − [(A_sample_ − A_blank_))/(A_control_ − A_blank_)]}

where, A_sample_ is the absorbance of the mixture (plant extract and DPPH), A_blank_ is the absorbance of the plant extract solution, and A_control_ is the absorbance of the ethanolic solution of DPPH.

Free radical scavenging at each concentration was repeated four times, and the data were expressed as the average ± the standard error of the means of the four experiments. Gallic acid was used as a positive control. The scavenging potential of the gallic acid and the various extracts was evaluated by the DPPH assay test and was expressed in terms of half-maximal effective concentration (EC_50_), where EC_50_ is defined as the concentration that causes a decrease in the initial DPPH concentration by 50%. The EC_50_ value was determined by extracting the equation for the linear part of the graph and substituting 50% for the *y* value, while calculating the concentration value of the *x*-axis. 

### 2.5. Antibacterial and Antifungal Activity

Microdilution tests were used to determine the MICs of the different samples. The broth microdilution assay was performed using twofold serial dilution in brain heart infusion (BHI) broth. The test was carried out in 96-well flat-bottomed microtitration plates. The cell suspension was prepared in BHI broth with an optical density equivalent to 0.5 of the McFarland standards, and diluted 1:100 in BHI broth to obtain a final concentration of 5 × 10^5^ colony-forming units per milliliter (CFU/mL). Controls with broth only and broth with bacteria without any of the antibacterial agents were also included in each plat. One hundred µl of the antibacterial agent were put in the first microplate well and serially diluted in BHI broth. One hundred µl, corresponding to 5 × 10^5^ CFU/mL, were added to all the wells. The plates were incubated at 37 °C for 18 h overnight. Erythromycin was used as a positive control for *S. aureus*, while nystatin was used as a positive control for *Candida albicans* and Tetracycline for *Salmonella typhimurium* strain LT2. The minimum inhibitory concentration (MIC) was defined as the lowest concentration able to inhibit the visible growth of bacteria in triplicate wells. After the MIC was visually determined, twenty microliters of *p*-iodonitrotetrazolium violet (8 mg/mL EtOH) were added to each well. The plate was incubated for another 30 min and assessed visually for any change in color from yellow to pink, which would indicate the reduction of dye due to bacterial growth.

### 2.6. Statistical Analysis

Statistical analyses were performed using Excel spreadsheet software (v16.0, Microsoft, Redmond, WA, USA). All experiments were conducted in quadruplicate, and the data were expressed as an average ± standard deviation. A *p*-value (calculated by *t*-test) of less than 0.05 was considered statistically significant.

## 3. Results and Discussion

**Figure 1:**
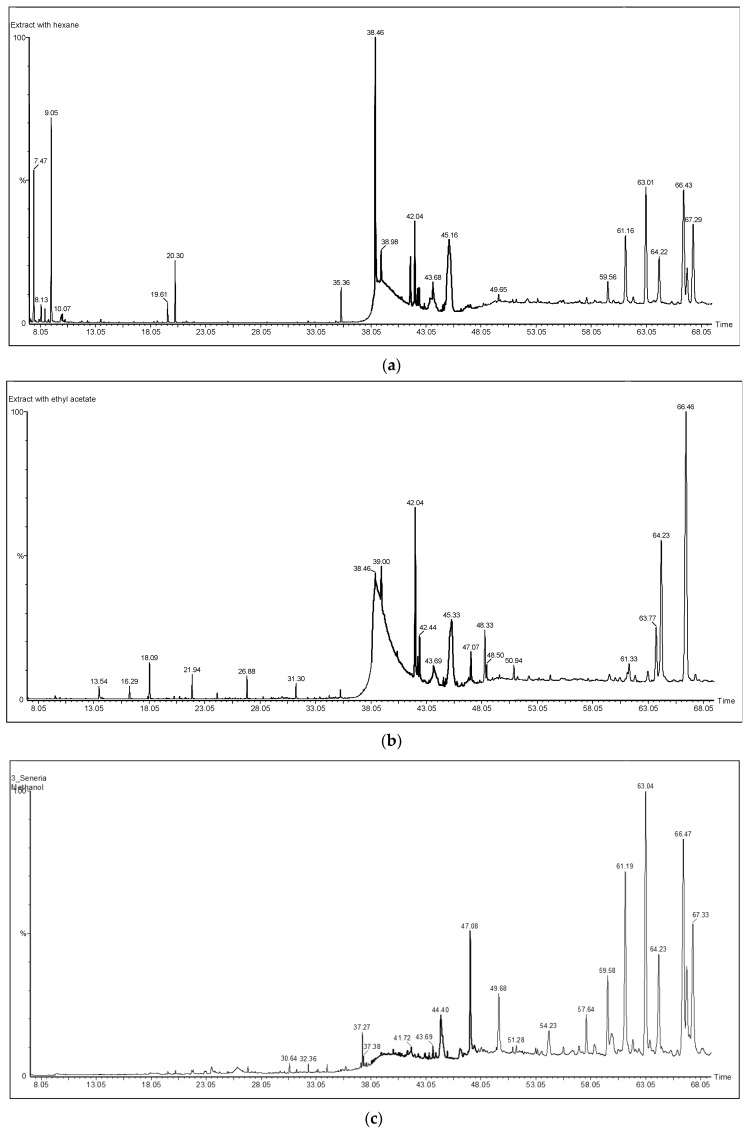
Typical GC-MS total ion chromatograms (TICs) of wild *Scolymus maculatus* extracts from *n*-hexane (**a**), ethyl acetate (**b**), and methanol (**c**).

**Figure 2:**
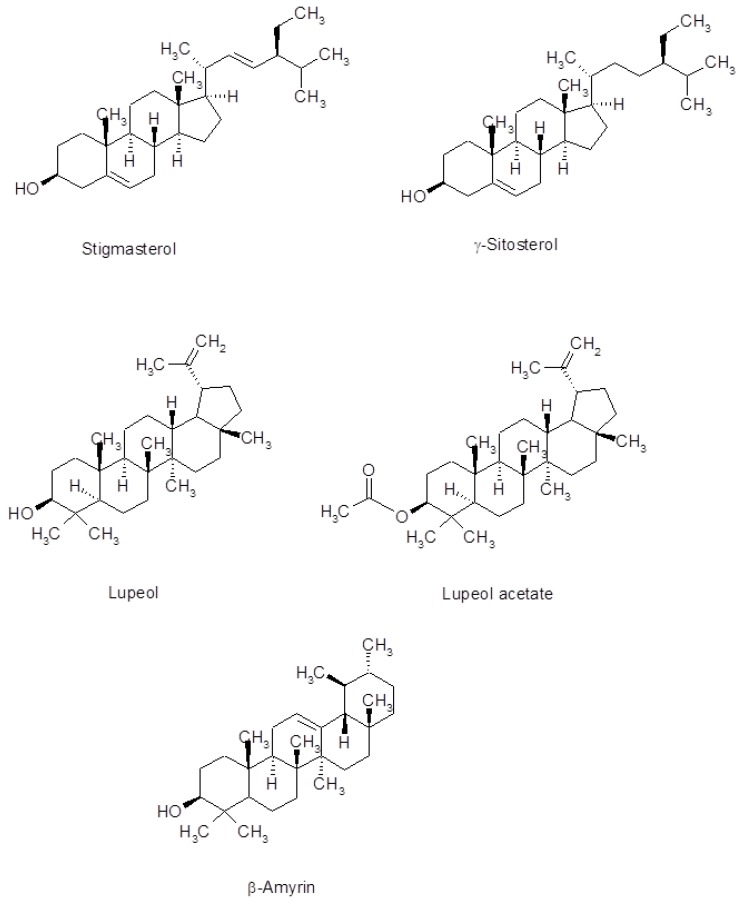
Phytochemicals stigmasterol, γ-sitosterol, lupeol, lupeol acetate, and beta-amyrin present in wild *Scolymus maculatus* extracts of methanol, ethyl acetate, and *n*-hexane, identified by GC-MS.

In this study, the phytochemical composition of wild *S. maculatus* methanol, ethyl acetate and *n*-hexane crude extracts was revealed. GC-MS screening in the electron impact mode (EI) revealed about 32 compounds for the first time. A satisfactory resolution and elution time were obtained on the capillary GC DB-5 column used (Figure 1). Of the 32 compounds seen in Table 1 and Figure 2, stigmasterol, γ-sitosterol, lupeol, lupeol acetate, and α-amyrin are known to exhibit important pharmacological activity, in particular anticancer, anti-inflammatory, and antibacterial activity [17,18,19]. Phytochemicals, such as 2-linoleoylglycerol (5.87%), γ-sitosterol (5.73%), β-amyrin (15.98%), lupeol (22.25%), (3α)-12-oleanen-3-yl acetate (9.14%), and lupenyl acetate (18.11%,) dominated the methanol extract (see Table 1 and Figure 1 and Figure 2). Almost all of these compounds were observed in ethyl acetate and *n*-hexane extracts, but in different proportions. 

It is worth noting that sitosterol has been reported to possess antibacterial potential [20]. Stigmasterol exhibits antibacterial activity against methicillin-resistant *Staphylococcus aureus* [16] and has been reported as well to significantly inhibit tumor promotion in two-stage carcinogenesis in mice [21]. A mixture of sitosterol and stigmasterol has been shown to possess anti-inflammatory activity after topical application [22]. Therefore, it is expected that the presence of such sterols in *S. maculatus* would be of paramount importance for combating and curing diseases.

Lupeol (Figure 2), another significant phytochemical in *S. maculatus*, is about 22% in the identified peaks in the methanol extract. Numerous preclinical animal studies suggest that lupeol has potential as anti-inflammatory, anti-microbial, anti-protozoal, anti-proliferative, anti-invasive, anti-angiogenic and cholesterol-lowering agent [23]. β-amyrin (Table 1 and Figure 2), which was found in the methanol and *n*-hexane extract in about 16% and 5.44%, respectively, were reported recently to have significant anticancer activity [24], and a recent study highlights the potential of both lupeol and stigmasterol as new, promising anti-MRSA (methicillin-resistant *Staphylococcus aureus*) agents [16]. β-amyrin from *L. microcladia* natural product has shown potential antibacterial activity [25]. The authors suggest intensifying the study on *L. microcladia* as a source of β-amyrin. Moreover, among the three molecules that were isolated from *S. globulifera*, lupeol and β-amyrin exhibit potential as new anti-*Enterococcus* compounds [26]. Growth inhibition of *Streptococcus* from the oral cavity by α-and β-amyrin terpenoids and α-amyrin esters have also been reported [27]. Table 1 shows the presence of α,β-unsaturated aliphatic aldehydes, such as 2,4-decadienal. Recent studies also demonstrate the capability of such a compound to combat bacterial growth [28,29].

**Figure 3:**
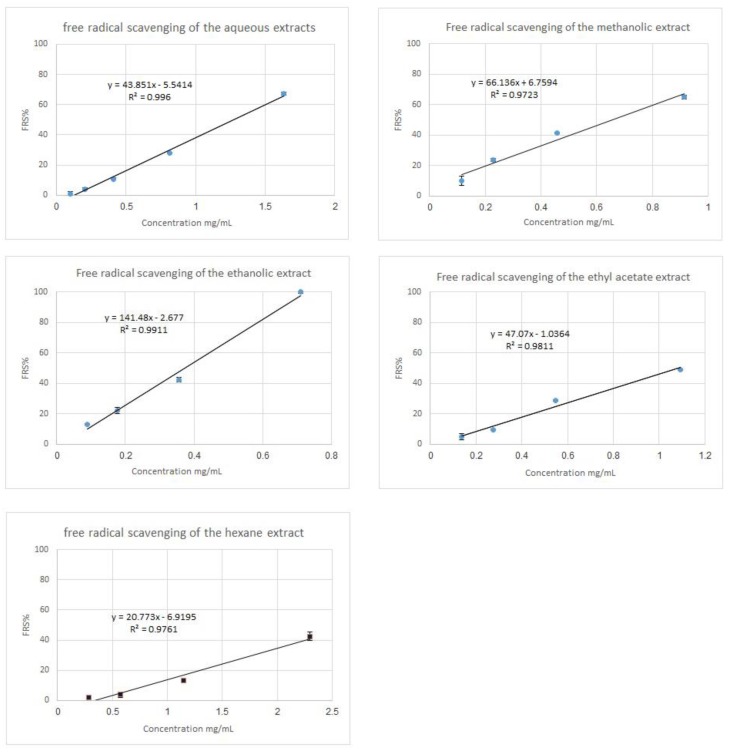
Free radical scavenging of all *S. maculatus* five extracts. At each concentration, the experiment was run four times. The data shown are the means ± the standard error of the means from the four experiments.

The antioxidant reacts with the stable free radical DPPH and converts it to 1, 1-diphenyl-2-picryl hydrazine. The ability to scavenge the free radical DPPH was measured at an absorbing wavelength of 517 nm. All the extracts of *S. maculatus* were tested for their free radical scavenging activity. The EC50 values of the five extracts are shown in Table 2 and Figure 3. The ethanolic extract is the most active (probably containing a higher concentration of antioxidants) while the *n*-hexane extract is the least active one. 

Antibacterial activity was anticipated because of the presence of compounds, such as lupeol, amyrin, sitosterol, and stigmasterol, which have shown potential antibacterial potential [20,25,30]. Therefore, the aqueous, methanol, ethanol, ethyl acetate, and *n*-hexane extracts of wild *S. maculatus* were evaluated in comparison to tetracycline, erythromycin and nystatin positive controls. The aqueous extract was deliberately used to imitate the usual method of plant decoction. The MICs against *Staphylococcus aureus, Salmonella typhimurium*, and *Candida albicans* microorganisms were determined. The ethyl acetate and the ethanolic extracts were active against the three types of microorganisms, with a MIC of 0.5 mg/mL, while the aqueous and the *n*-hexane extracts were inactive against *Salmonella typhimurium* (Table 3). The methanolic extract was active against *Staphylococcus aureus* and *Candida albicans* but not against *Salmonella typhimurium*. 

## 4. Conclusions

Thirty-two volatile components were detected in wild *S. maculatus* for the first time. Lupeol was the predominant component, with a percentage exceeding 22% in methanol. Lupeol acetate was the principle phytochemical in the ethyl acetate extract (26%) and to a lesser extent (11%) in *n*-hexane. Stigmasterol, γ-sitosterol, lupeol, lupeol acetate, and β-amyrin, which are reported here for the first time as phytochemical components in *S. maculatus,* are biologically active compounds according to scientific literature and most likely responsible for some of the activities of wild *S. maculatus*. Isolation of all antioxidant and antimicrobial chemicals from the most active extracts and testing their contribution to the biological activities of wild *S. maculatus* and potential synergism is recommended. The findings disclosed herein are important and may suggest the importance of consuming *S. maculatus* as food. Enriched extracts could be prepared that possess certain activity beneficial to human health, and in a broader context, that could be useful for the treatment of various diseases. As well, *S. maculatus* could be a source of promising lead compounds for the development of new treatments for microbial-related diseases and some other diseases. 

## Figures and Tables

**Table 1:** Phytochemical percentages and retention times for wild *S. maculatus* extracts of methanol, ethyl acetate, and *n*-hexane, identified by gas chromatography-mass spectrometry (GC-MS).

Phytochemical Names and Retention Times				Area %	
No.	Compound Name	RT (min)	Methanol	Ethyl Acetate	*n*-Hexane
1	1-Ethylbutyl hydroperoxide	9.054			6.42
2	1-Ethyl-2-heptylcyclopropane	16.29		0.32	
3	Acetoglyceride	18.09		1.19	
4	2,4-Decadienal	20.303			1.78
5	Nonylcyclopropane	21.89		0.63	
6	(E)-3-Octadecene	26.88		0.59	
7	Coniferyl alcohol	30.64	0.37		
8	6,10,14-Trimethyl-2-pentadecanone	32.36	0.24	0.40	
9	Ethyl palmitate	35.36			0.99
10	Methyl linoleate	37.27	0.97		
11	14-Octadecenoic acid, methyl ester	37.38	0.30	0.24	
12	Ethyl linolelaidate	38.455			12.48
13	Ethyl (9E)-9-octadecenoate	38.554			2.35
14	Palmitamide	39.00		42.67	11.97
15	Doconexent	40.08	0.69		2.61
16	Oleamide	42.04		5.08	2.90
17	Octyl cyclohexanecarboxylate	42.44		1.27	0.74
18	Tetracosane	44.4	2.15	0.28	
19	Diploptene	45.16			15.68
20	2-Linoleoylglycerol	47.08	5.87	1.46	0.39
21	Spinacene	48.49		0.47	0.32
22	Tetratriacontane	49.68	2.34		1.01
23	Unknown	54.23	2.04		
24	Stigmasterol	57.64	2.55		0.27
25	γ-Sitosterol	59.58	5.73	0.22	1.59
26	β-Amyrin	61.19	15.98	0.61	5.44
27	Stigmasterol acetate	61.33		1.27	
28	Lupeol	63.04	22.25		10.05
29	Stigmastan-3,5-diene	63.76		4.21	
30	(3α)-12-Oleanen-3-yl acetate	64.23	9.14	12.11	4.22
31	Lupenyl acetate	66.47	18.11	26.82	11.03
32	Unknown	67.33	11.26	0.16	7.78

**Table 2:** The half-maximal effective concentration (EC_50_) values of the radical scavenging activity of five *S. maculatus* extracts: aqueous, methanolic, ethanolic, ethyl acetate, and *n*-hexane (*n* = 4).

*S. maculatus* Extract Type	EC_50_ (mg/mL)
Aqueous extract	1.27
Methanolic extract	0.65
Ethanolic extract	0.37
Ethyl acetate extract	1.08
*n*-hexane extract	>2.0

**Table 3:** The minimum inhibitory concentrations (MICs) of five *S. maculatus* extracts (aqueous, methanolic, ethanolic, ethyl acetate, and *n*-hexane) against *Staphylococcus aureus*, *Salmonella typhimurium*, and *Candida albicans* microorganisms.

Type of Extract/Microbial Strain	*Staphylococcus aureus*	*Salmonella typhimurium*	*Candida albicans*
Aqueous extract	0.5 mg/mL	>4.0 mg/mL	1.0 mg/mL
Methanolic extract	0.5 mg/mL	>4.0 mg/mL	0.5 mg/mL
Ethanolic extract	0.5 mg/mL	0.5 mg/mL	0.5 mg/mL
Ethyl acetate extract	0.5 mg/mL	0.5 mg/mL	0.5 mg/mL
*n*-hexane extract	1.0 mg/mL	>4.0 mg/mL	0.5 mg/mL
Tetracycline	-	0.01 mg/mL	-
Erythromycin	0.0078 mg/mL	-	-
Nystatin	-	-	0.00312 mg/mL

## Abbreviations

DPPH	diphenylpicrylhydrazyl
AA	antioxidant activity
MIC	minimum inhibitory concentration
GC-MS	gas chromatography-mass spectrometry
EI	electron impact
BHI	brain heart infusion
EC_50_	half-maximal effective concentration
TIC	total ion chromatogram
